# Public health triangulation: approach and application to synthesizing data to understand national and local HIV epidemics

**DOI:** 10.1186/1471-2458-10-447

**Published:** 2010-07-29

**Authors:** George W Rutherford, William McFarland, Hilary Spindler, Karen White, Sadhna V Patel, John Aberle-Grasse, Keith Sabin, Nathan Smith, Stephanie Taché, Jesus M Calleja-Garcia, Rand L Stoneburner

**Affiliations:** 1 Global Health Sciences, University of California, San Francisco, California, USA; 2 the San Francisco Department of Public Health, San Francisco, California, USA; 3 the Global AIDS Program, Centers for Disease Control and Prevention, Atlanta, Georgia, USA; 4 the Public Health Prevention Service, Epidemiology Program Office, Centers for Disease Control and Prevention, Atlanta, Georgia, USA; 5 the HIV/AIDS Division, World Health Organization, Geneva, Switzerland; 6 ST: Institut für Allgemein-, Familien- und Präventivmedizin, Paracelsus Medizinishce Privatuniversität, Salzburg, Austria, and RLS: the Joint United Nations Programme on HIV/AIDS in Geneva, Switzerland

## Abstract

**Background:**

Public health triangulation is a process for reviewing, synthesising and interpreting secondary data from multiple sources that bear on the same question to make public health decisions. It can be used to understand the dynamics of HIV transmission and to measure the impact of public health programs. While traditional intervention research and metaanalysis would be ideal sources of information for public health decision making, they are infrequently available, and often decisions can be based only on surveillance and survey data.

**Methods:**

The process involves examination of a wide variety of data sources and both biological, behavioral and program data and seeks input from stakeholders to formulate meaningful public health questions. Finally and most importantly, it uses the results to inform public health decision-making. There are 12 discrete steps in the triangulation process, which included identification and assessment of key questions, identification of data sources, refining questions, gathering data and reports, assessing the quality of those data and reports, formulating hypotheses to explain trends in the data, corroborating or refining working hypotheses, drawing conclusions, communicating results and recommendations and taking public health action.

**Results:**

Triangulation can be limited by the quality of the original data, the potentials for ecological fallacy and "data dredging" and reproducibility of results.

**Conclusions:**

Nonetheless, we believe that public health triangulation allows for the interpretation of data sets that cannot be analyzed using meta-analysis and can be a helpful adjunct to surveillance, to formal public health intervention research and to monitoring and evaluation, which in turn lead to improved national strategic planning and resource allocation.

## Background

Public health practitioners are frequently faced with having to make programmatic decisions with less than perfect data. It is seldom the case that a high-quality study exists that can provide answers to everyday public health questions directly, and often we must rely upon imperfect surveillance data to guide policy and programmatic decisions. This has been particularly the case for HIV, where the urgency of the epidemic coupled with enormously scaled-up international resources demand rapid responses in planning and targeting prevention and care programs. Further, the last several years have seen a massive increase in not only HIV surveillance data but also in programmatic data, quantitative and qualitative research studies, and local and national experience and expertise. While the volume of information has increased exponentially, its use and analysis to inform program, planning and policy have been lagging. Diverse sources of data are rarely presented together, and gathering, synthesizing and interpreting them has become increasingly challenging.

"Triangulation" is a term that can broadly refer to an approach to synthesizing multiple, diverse sources of data at the level of interpretation. However, the word itself has come to mean different things in different fields. Social scientists have used the term "triangulation" since the late 1960 s [1]. By triangulation they mean examining multiple data sources to validate results, increase credibility and gain a more detailed understanding of findings [2,3]. The term has been used to refer to methods for establishing both internal and external validity by decreasing the uncertainty of a single measurement by making multiple observations [4]. Denzin describes four types of triangulation: data triangulation, in which data gathered through different samples and at different times are compared; investigator triangulation, in which more than one investigator examines the same question and results are compared; theory triangulation, in which different theoretical constructs are applied to the same observed data; and method triangulation, in which phenomena are examined using different methods [5]. The term triangulation has also been used in nursing research to refer to mixed qualitative and quantitative methods to explain complex phenomena [6-8], akin to Denzin's method triangulation. In these contexts, "triangulation" often implies that the approach was created at the design stage, where the investigator could exert control over the study methods and measures. Triangulation is similar to evidence mapping [9,10], and realist synthesis [11,12]. These, however, tend to be more forward-looking and less concerned about explaining what has transpired in the past, creating the present day situation. It is also similar to narrative reviews but tends to have a broader focus than individual interventions [13]. Triangulation, as used in the context of public health, is probably most closely related to critical interpretive synthesis, which has as its focus the generation of theory to interpret observations and is not nearly as methodologically constrained as meta-analysis [14]. However, as discussed below, triangulation extends the construct of critical interpretive synthesis by addressing not only how we got to where we are but also what we do next.

More recently the term has been used in the context of public health to refer to the process of reviewing and interpreting secondary data from multiple data sets that bear on the same question to make public health decisions, combining elements of both data triangulation and method triangulation [15,16]. The work of Stoneburner and Low-Beer in Uganda offers an early example of this approach to triangulation [17]. Their basic question was whether or not the declining trends in HIV prevalence seen in women attending antenatal clinics in Kampala and elsewhere in the early to mid 1990 s were the result of declines in HIV incidence or the result of mortality. The scope of the question and the timeframe encompassed did not allow for the design of a single prospective study that could answer this question directly and definitively. The changes during this period were substantial with HIV prevalence falling from 21.1% to 9.8% between 1991 and 1998 and to 6.4% in 2001 [18] based on data from antenatal clinic sites. They were able to confirm similar trends in other national datasets, such as a decline in HIV prevalence in male army recruits and blood donors and declines in directly measured HIV incidence in large cohort studies in the country [19]. They also identified a series of data sets that bore on variables in the chain of events that led to prevalent HIV infection in women, including age at sexual debut, risk factors for exposure to HIV (men's and women's numbers of partners, communication about avoidance of risk), risk factors for transmission (use of condoms), incident HIV infection and mortality. They were able to show that an increase in age of sexual debut and decreasing numbers of sexual partners, which would have led to contraction of sexual networks, temporally preceded the decline in HIV prevalence [13]. Moreover, data from neighboring countries with HIV epidemics in a similar stage as Uganda did not show comparable declines in early sexual debut and number of sex partners nor subsequent declines in HIV prevalence, further suggesting that behavior change rather than mortality was the cause of the decline in HIV prevalence seen in Uganda [20]. Additionally, prevention programs at the time emphasized partner reduction or "zero grazing". The success of Uganda was heralded as evidence that national level responses could produce sweeping impact on the HIV epidemic. In this example, we highlight the success of the triangulation approach in assembling and interpreting diverse existing data sources to provide evidence in support of the underlying cause of the national epidemic trend. There are several other examples where public health triangulation has been used to understand trends, for instance, from Cambodia, Thailand and the United States [21-25], and the 5-year evaluation of the Global Fund to Fight AIDS, Tuberculosis and Malaria [26] is using not dissimilar methods

In this paper we propose a standard approach to public health triangulation and suggest scenarios in which it can enhance understanding of the national and local HIV epidemics and prove useful in programmatic decision-making. We define public health triangulation as the process of reviewing and interpreting existing data and trends in those data from multiple data sources that bear on different facets of a broad public health question in order to identify factors that underlie the observed data and to assist with public health decision-making and actions.

## Methods

### Approach to Public Health Triangulation

We outline five guiding principles of public health triangulation:

1. Use of existing data; on occasion very limited new data abstraction or cross-matching between data sets;

2. Synthesis of data qualitatively, similar to what is done in a narrative review, in contrast to combining data sets quantitatively for statistical analysis;

3. Inclusion of diverse data sources, such as surveillance, research, programmatic and expert opinion; both quantitative and qualitative data; and both biological and behavioral measures;

4. Input from stakeholders for the formulation of a public health question, data identification and assessment of data, interpretation and dissemination of results;

5. Using results to inform public health decision-making

While the main goal of triangulation is to inform public health decisions, triangulation can also be used to assess the external validity of observed trends as well as to examine the effectiveness of widely disseminated interventions on multiple outcomes, such as knowledge, attitudes, behaviors and actual disease incidence, at the population level. Whereas traditional intervention research and systematic reviews seek to answer pre-formed hypotheses, triangulation seeks to strengthen interpretations and improve decisions based on the available evidence. Triangulation does not formally demonstrate causality in the same manner as a purposefully designed randomized controlled trial but rather offers a rational explanation or interpretation of the data at hand.

Given the diverse uses of the word "triangulation" and its broad applicability, it may be useful to say what public health triangulation is not in our current methodological framework. First, it is not conventional meta-analysis. Meta-analysis combines methodologically similar data sets with similar outcome and predictor variables at level of statistical analysis, whereas public health triangulation examines methodologically dissimilar data and whether they corroborate each other. Secondly, public health triangulation is not a systematic review of the published literature. In addition to quantitative and qualitative data sources, it involves extensive searching of the unpublished reports and uses programmatic data, unpublished data sets and expert opinion as well as published studies, meta-analyses and systematic reviews. Thirdly, in our approach, public health triangulation does not involve primary data collection. Although occasionally the situation has presented itself where data need to be abstracted from written records or where we were able to match patients from one data set to another, such as linking AIDS and sexually transmitted disease cases in San Francisco [27], this is more the exception than the rule. Fourthly, it is also not a method to evaluate the performance of a newer data gathering method against an established "gold standard". Finally it is a not a technique for rapid assessment, such as rapid assessment and response (RAR) [28], that involve assessments in a microenvironment with data collected prospectively as well as retrospectively. These methods are different from the process of public health triangulation we describe here that involves trends in large geographical areas and rarely employ prospective data gathering. Additional differences between public health triangulation, as we have applied it, and conventional epidemiologic research are shown in Table 1.

**Table 1:** How public health triangulation differs from conventional epidemiologic analysis

Public health triangulation analysis	Conventional epidemiologic analysis
Inductive, empirical	Deductive

Emphasis on 'best possible' existing data	Emphasis on data of highest scientific rigor

Focus on plausibility as basis for conclusions (with or without statistics)	Focus on statistics as basis for conclusions

Focus on external validity: *"Can observed effects be generalized to the larger population?"*	Focus on internal validity: *"Did A cause B in our study?"*

Based on inter-connected pieces of the same situation	Based on independent samples

Qualitative interpretation	Mathematical modeling

Goal: public health decision-making	Goal: increasing scientific knowledge

Data triangulation and synthesis activities in general are key components for monitoring HIV epidemics and should be incorporated into routine monitoring and evaluation systems. Most countries already conduct these activities at some level; an example is developing an epidemiological profile as the first part of a national prevention plan. In addition, these activities are part and parcel of several focused efforts including the World Health Organization's (WHO) Second-Generation Surveillance [19], the Policy Project's A-Squared: Analysis & Advocacy [29], UNAIDS and World Bank's Modes of Transmission Study [30], and UNAIDS' "Know Your Epidemic" [31]. All these activities tap into similar data sources and share some objectives; however, they are more heavily focused on understanding HIV incidence and prevalence, use specific arrays of data and often involve modeling. In contrast, public health triangulation uses a different approach and supports the added dimensions of stakeholder-driven processes for generating key questions, identifying relevant existing data sources, formulating hypotheses and interpreting and using data.

## Results

### The public health triangulation process

Public health triangulation is an iterative process in which key questions and hypotheses that potentially explain them are formulated, examined and reexamined as additional data become available. It can be conducted at the national or sub-national level and can be done once or repeated serially over time. Triangulation can be broken down into a series of forward-moving steps (see Table 2) that start with framing key questions and end with plans for public health action. Local stakeholders, key institutions or persons that have a vested interest in how the response to the HIV/AIDS epidemic is directed and how the data are being used, drive much of the process of triangulation. Moreover, stakeholders' participation is key to success because access to data sets will most often depend on them. Triangulation is most successful when stakeholders from multiple disciplines and representing multiple interested agencies are involved in all phases, including deciding the priority questions to be answered, identifying and gathering data, guiding the process of data review and interpretation, and using the results of the triangulation in their own policy and program decision-making.

**Table 2:** The public health triangulation process.

1. Identify key questions
2. Ensure question(s) are important, actionable, answerable and appropriate for triangulation
3. Identify data sources and gather background information
4. Refine the questions
5. Gather data/reports
6. Assess data reliability and make observations from each data set
7. Note trends across data sets and hypothesize
8. Check (corroborate, refute, modify) hypotheses
9. If necessary, identify additional data and return to Step 5
10. Summarize findings and draw conclusions
11. Communicate results and recommendations
12. Outline next steps for public health action

### Framing a question

The first step in conducting public health triangulation is to choose a key question that is suitable for the method (Table 3). Triangulation exercises usually and logically begin with very broad questions: Is the HIV epidemic increasing, decreasing, or stabilizing nationally? Why is HIV prevalence different in different parts of a country? What are the local drivers of the HIV epidemic? How important are most at-risk populations, such as men who have sex with men, injection drug users and female sex workers in local epidemic dynamics? What has the impact of antiretroviral therapy (ART) on HIV incidence or mortality been? Questions need to be sufficiently important (i.e., address a large part of the epidemic) to justify the in-depth search for data, to be actionable (i.e., that an answer will result in public health decisions), to have sufficient data available to answer the question and to be feasible given time and resource constraints. Questions selected for triangulation should not include those better addressed by traditional study methods, such as randomized controlled trials or cohort studies. After addressing questions at a national level, specific questions, such as local differences in factors driving transmission or the impact of specific prevention approaches can be addressed at later stages.

**Table 3:** Criteria for a question answerable by public health triangulation

Public health importance	Is the question being asked of sufficient public health importance to justify the investment of human resources and funding? [e.g., should a question regarding the impact of rare injection drug use in an African country be pursued or one focused on much more prevalent alcohol use?]
Actionable	Will answering the question being asked lead to the initiation of a public health action? Will the risk factors we identify be modifiable and amendable to public health interventions?
Data availability	Are there data available that have asked the right questions and provide answers on the different steps in the sequence of events that leads to a public health outcome?
Appropriateness	Can the question be answered with conventional research methods or with a single available data set? Is a proposed intervention sufficiently new and unique that it should be evaluated by a different methodology?
Feasibility	Are there sufficient human resources and funding available to gather and analyze the data? Unless sufficient resources are available, searching for data and conducting the multiple levels of analysis needed for triangulation may be inadequate

Conceptually, the selection of a public health triangulation question is two-step: first focusing on whether the question is important and actionable (broad policy considerations) and second on the logistical considerations. Logistical considerations can often be addressed with more detailed fieldwork, whereas if the broad policy considerations are not met, the field effort may not be worthwhile. The process should guide stakeholders towards questions that are feasible, actionable and most amenable to public health triangulation. Note that the focus of these questions should be on identifying modifiable risk factors that are amenable to public health interventions rather than on non-modifiable risk factors, such as age or sex. Frequently, this helps prioritize questions to be examined. For example in Malawi, stakeholders identified 33 questions initially; two were chosen after extensive discussion:

▪ Has HIV prevalence (incidence) increased, decreased or remained the same in Malawi from 2000 to 2005 and has this change been due to a modifiable risk factor?

▪ What was the reach and intensity of HIV prevention programs in Malawi from 2000 to 2005?

These two key questions were eventually selected based on how actionable the answers might be (scored as high, medium and low) and what data sources were available. In contrast, for example, one proposed question, "What is the relationship between alcohol and risk behavior?" received lower priority. Although alcohol use is important and potentially actionable, clear interventions to reduce alcohol-related transmission have not been successful in the context of generalized epidemics. Moreover, there were only a few questions from prior Demographic and Health Surveys that could have provided data.

### Understanding the sequence of events that leads to an outcome

In order to posit a causal association between a specific modifiable variable (e.g. multiple partners) and an outcome (e.g., HIV incidence), among other criteria, changes in trends for the variable should temporally precede changes in the outcome. This is a necessary but not sufficient criterion for assessing causality. The most useful data for triangulation are therefore those that describe trends in predictor and outcome variables that can logically show the sequence of events. For instance, a variety of behaviors and biological events, such as early age at first intercourse, multiple concurrent sexual partners, having ever paid for sex, not using condoms, having had a sexually transmitted infection recently and not being circumcised, are all risk factors for either exposure to HIV or, if exposed, sexual transmission of HIV. Changes in these variables should therefore logically precede or coincide with changes in HIV prevalence or incidence. Moreover, these examples are all modifiable risk factors, as opposed to non-modifiable risk factors such as age or sex. For example, in Uganda declines in the numbers of non-regular partners among men temporally preceded declines in HIV prevalence among women seeking antenatal care [17]. Additionally, other variables may modify these risks and need to be considered on the causal pathway. An example might be factors that protect against exposure, such as an infected sexual partner's low viral load, or if exposure occurs, against transmission, such as circumcision.

### Data gathering

As with conventional systematic reviews and meta-analysis, searching for evidence is the most important task in public health triangulation. Once identified, all relevant data and information are initially considered. Typically, data originate from public health and household surveillance, national censuses, public health programs and smaller epidemiologic research studies that often appear only in abstract form or are presented at regional or national meetings. These data are identified through conventional data searches and with the assistance of ministries of health, non-governmental organizations and local academic institutions, which are typically represented among the stakeholders, and in our experience, which has primarily focused on HIV/AIDS in low- and middle-income countries, there are large amounts of data available. Both quantitative and qualitative data are useful, and understanding of the local context in which one is trying to answer a question is often enriched with qualitative data rather than relying completely on quantitative data. Moreover, local knowledge and expert opinion are particularly important in understanding potential relationships between predictor and outcome variables. Identifying and accessing the relevant data are requisite steps in being able to use these methods.

### Assessing data quality and reliability

Some data sources are better than others in the sense that those generating the data have taken steps to minimize bias, control for confounding and maximize the likelihood of generalizability and external validity. For example, HIV surveillance data are often a core data source for triangulation, so sampling frameworks, selection of participants, underreporting and non-response bias are important considerations when assessing representativeness. While we can use formal quality criteria for observational and experimental studies [32,33], we are more frequently faced with assessing quality of survey data for which formal evaluation criteria have not been published. In lieu of formal quality criteria for surveys and surveillance studies, the following questions, largely bearing on the representativeness of the sample and the generalizability of the results, can be used to assess external validity:

• From where was the sample selected and why?

• Who was selected and why?

• How were they selected and why?

• Was the sample size justified?

• Is it clear why some participants chose not to take part?

Classifying findings from each data source according to their quality can be helpful in understanding contradictory results. While there are many reasons why studies may have different results, one important reason is that poorly conducted studies are more prone to error than well-conducted studies. Parenthetically, we recognize the difficulties in using qualityscoring scales for studies; differences in reported quality may be as much a function of poor reporting as a poorly conducted study [34]. However, as a general rule if higher-quality studies have findings that differ from those in lower-quality studies, we give greater weight to the findings from the higher-quality studies. We emphasize that it is important to understand all findings, and care should be exercised to not dismiss contradictory results out of hand, even if they are of lower quality. Nonetheless, in general the highest quality data should be arrayed and interpreted first and given priority. Guidelines for assessing the reliability of different types of data are outlined in Table 4.

**Table 4:** Criteria for ranking data sources.

Data type	Criteria
**Program data**	• Uniform data collection and reporting tool used?
	• Frequency and timeliness of reporting
	• Adherence to a standard operating procedure for data management?
	• Data coverage (Number of sites included in the reporting system/total number of sites offering services)
	• Sample size (patient, client, or product (i.e. condoms))

**Surveillance data (ANC, DHS, BSS, etc.)**	• Representativeness of sample for the target population (probability based?)
	• Implementation (implemented according to protocol)
	• Strength of the measures (biomarkers, self report, detailed behavioral indicators, knowledge)
	• Analysis (appropriate, complete, methods detailed)
	• Frequency and timeliness of data collection and reporting
	• Consistency of sites/locations and populations measured over time
	• Sample size
	• Response rate

**Special studies (qualitative)**	• Sampling strategy explained in detail
	• Interview/observation methods described in detail
	• Implementation (methods implemented according to protocol)
	• Analysis (appropriate, complete, methods detailed)
	• Sample size

**Special studies (quantitative)**	• Representativeness of sample (probability based?)
	• Implementation (methods implemented according to protocol)
	• Strength of the measures (biomarkers, detailed behavioral indicators, knowledge)
	• Analysis (appropriate, complete, methods detailed)
	• Sample size
	• Response rate

Many of the data sets we have dealt with address trends over time. Thus, consistency of sampling and data collection methods and sufficient sample size to detect significant trends are important as well. For instance, an HIV sentinel survey of prepartum women attending antenatal clinics should ideally use the same clinics from year to year or at least present data in a way that we can ascertain trends in the clinics that have been surveyed longest and most consistently.

### Formulating and assessing hypotheses

At its core public health triangulation seeks to confirm or refute explanatory hypotheses with available data. This process starts with the key questions and review of data by person, place and time for each data set using tables, figures and maps. We then examine trends in key indicators (both predictor and outcome variables) to see if there is a logical temporal and spatial relationship among trends in one or more indicators (predictor variables) and outcomes and across multiple data sets. Ideally, we can corroborate the same or similar indicator trends in different data sets (e.g., a decline in HIV prevalence in women attending ANC, a decline in HIV prevalence in military recruits, a decrease in HIV prevalence in two Demographic and Health Survey with HIV testing (DHS+ surveys). Second, we seek to corroborate different indicators (e.g., a decrease in HIV prevalence, a decrease in STI, a decrease in multiple partners) in the same data set and across data sets. Third, we attempt to corroborate and further our understanding of the trends in the indicators with other information, including qualitative studies, expert opinion and programmatic data, for example. If the same conclusions are reached across the diverse data sources, different indicators and mix of methods, we have successfully "triangulated" a finding in the social science sense of using multiple data sources to externally validate a finding. If we the data sources lead in different directions, then we attempt to understand why, for instance, by examining for modifying variables, different sampling methods or different study quality.

We next formulate possible hypotheses that explain the data and can potentially answer the original key question, with particular attention to data trends that we have collected and reviewed. This process is inductive and empirical; we seek to identify hypotheses that explain all the data at hand and suggest a causal relationship between predictor variables (e.g., a public health intervention) and outcomes (e.g., risk behaviours or infection). It is very important to not discard data that do not fit a hypothesis. All data have to be accounted for or explained, and, while often a perfectly reasonable explanation is that an outlier was derived from a methodologically flawed study, outliers need to be examined closely before being excluded on methodological grounds alone. If an explanatory hypothesis fits all the data, it is confirmed; if not, we may need to gather additional data or reformulate the hypothesis. Epidemiologic criteria, such as those proposed by Hill [35], for assessing if the relationship between predictor and outcome variables is potentially causal can be especially helpful to assist in formulating hypotheses.

Triangulation is an iterative process. Refining hypotheses to better match the data is central to public health triangulation. We are searching for the hypothesis or explanation that is consistent with the preponderance of the data and is biologically or socially plausible. If the weight of the evidence is inconsistent with the hypothesis, the hypothesis should be rejected. It is important to mention that the findings from this data review process are not as dependent on statistical testing as in traditional experimental or observational studies. Triangulation results are based on repetition of findings from multiple data sources, often using findings derived from non-identical methodologies.

### Drawing conclusions

Accepting an hypothesis as the best answer to the question that was originally asked is based on the preponderance and strength of the evidence. When comparing alternative hypotheses, we ask:

• Which hypotheses explain all or most of the data? Which are supported by the most independent sources of data?

• Which hypotheses are supported by the most rigorous data, the best study designs and the most generalizable results?

• Which hypotheses meet the most epidemiologic criteria for causality, especially biological or sociological plausibility?

• Which hypotheses are supported by both the 'numbers' and the 'stories' (quantitative, qualitative and expert opinion)?

• Would the likely biases, limitations and potential confounders in one or more studies in the analysis change conclusions?

• Have all the alternative explanations been considered and rejected?

For example, in Mozambique prevalence of HIV in women attending antenatal clinics is substantially lower in the northern three provinces than in the rest of the country [36]. Behavioral data from prior surveys indicate that sexual risk is just as high as in the rest of the country and condom use, if anything, is lower. However, male circumcision rates are far higher than in the rest of the country approaching 80% or more. Thus, we can suggest that male circumcision, which has been shown to be effective in three randomised controlled trials in other countries of the region, may be causally associated with the low prevalence. The public health action is to suggest male circumcision programs for other higher prevalence parts of the country and to evaluate its impact.

## Discussion

Stated another way, triangulation attempts to use the preponderance of the evidence collected to support the hypotheses, with particular attention paid to trends and consideration given to both supporting and refuting findings. Triangulation can be thought of like detective work, each new clue or data point can serve to confirm or refute your working hypothesis, or to lead you in search of additional data or alternative explanations. There are often multiple ways to arrive at the answer. Just like a good detective, a good practitioner of public health triangulation develops experience over time, which can allow him or her to develop narrower and better hypotheses, and know where to look for the best 'clues'. Often the best clues come from stakeholders who know the situation best; finding the right answer to questions is empirical, and initial ideas that lead to hypotheses are frequently best informed by stakeholders with an experiential sense of the social dynamics of HIV transmission in local populations.

### Limitations of public health triangulation

Public health triangulation has several limitations. First are the data on which it is based. Data that directly answer the question being asked are not likely to be found, and inferences have to be made based on available data. These are often problematic: How free of bias are they? How likely are they to be externally valid? We have found that identifying and examining data that bear on different points in the chain of events that leads to the outcome in question is useful for strengthening the likelihood that the individual trend in question, such as declining HIV prevalence among women attending antenatal clinics in Uganda, is part of a larger series of events that has culminated in the observed trend.

Secondly we risk ecological fallacy. We often are faced with the situation in which we have trend data for predictor variables and trend data for outcome variables, but the predictor and outcome variables are not linked at the level of the individual survey participant. Thus, our interpretations may be the consequence of ecological fallacy if we are not able to refer to an underlying model that lays out how predictor variables are associated with outcome variables. We have found applying Hill's criteria [35] useful in aiding our understanding of the potential causal relationships between trends in predictor variables and trends in outcome variables. In fact, our framework for public health triangulation echoes one of Hill's criteria, consistency of results in several studies of different design.

Thirdly we face the problem of "data dredging"; that is, analyzing large data sets without null hypotheses and often failing to correct statistical tests for multiple comparisons. To guard against this, our practice is to be scrupulously aware of all the data at hand and only reject non-supportive data with great reluctance and for clear reasons. Additionally, the recommendation to conduct all phases of triangulation with the body of stakeholders is in part to prevent the preconceptions of one or a few persons from dominating the process.

Fourthly, we are concerned about the reproducibility of results. Different investigators should be able to come up with the same answer to a question that has been posed. We believe that reproducibility lies in a careful search for available data, careful scrutiny of the data themselves and neither over interpreting the data at hand nor ignoring data that do not fit hypotheses.

## Conclusions

Public health triangulation is a process for the synthetic interpretation of data sets that either cannot be matched at the individual patient level or combined in meta-analysis; it can be a helpful adjunct to surveillance, to formal public health intervention research and to monitoring and evaluation (for examples, see [37,38]) While many public health practitioners instinctively compare data from different sources to ascertain whether observed trends are consistent biologically and sociologically and mutually reinforce each others' external validity, we believe that a more formal examination of all available data sets, which often do not include those routinely examined by national AIDS control programs, such as vital statistics and data from other sectors like the military and police, can enhance the understanding of what is happening epidemiologically in a country. More usefully perhaps, the process of public health triangulation can also examine the impact of interventions, such as in Uganda, and suggest geographical areas and populations in need of interventions. This is perhaps public health triangulation's greatest utility.

Public health triangulation should not displace formal evaluation of interventions, carefully constructed surveillance systems or formal monitoring and evaluation activities, but it does offer an opportunity to compare and contrast the data generated by these activities with the end of improving public health outcomes. We believe that public health triangulation can be of assistance to the practitioner who needs to make public health decisions - allocating resources, initiating control programs, deciding to expand or discontinue specific prevention activities - and to evaluate their impact in real time.

## List of Abbreviations

ANC: antenatal clinic; ART: antiretroviral therapy; BSS: behavioral surveillance system; DHS+: Demographic and Health Survey plus HIV testing; HIV: human immunodeficiency virus; RAR: Rapid Assessment and Response; UNAIDS: Special United National Programme on HIV/AIDS; WHO: World Health Organization

## Competing interests

The authors declare that they have no competing interests.

## Authors' contributions

GWR made substantial contributions to conception and design, or acquisition of data, or analysis and interpretation of data; was involved in drafting the manuscript or revising it critically for important intellectual content; and gave final approval of the version to be published. WMcF made substantial contributions to conception and design, or acquisition of data, or analysis and interpretation of data; was involved in drafting the manuscript or revising it critically for important intellectual content; and gave final approval of the version to be published. HS made substantial contributions to conception and design, or acquisition of data, or analysis and interpretation of data; was involved in drafting the manuscript or revising it critically for important intellectual content; and gave final approval of the version to be published. KW made substantial contributions to conception and design, or acquisition of data, or analysis and interpretation of data; was involved in drafting the manuscript or revising it critically for important intellectual content; and gave final approval of the version to be published. SVP made substantial contributions to conception and design, or acquisition of data, or analysis and interpretation of data and gave final approval of the version to be published. JA-G made substantial contributions to conception and design, or acquisition of data, or analysis and interpretation of data; was involved in drafting the manuscript or revising it critically for important intellectual content; and gave final approval of the version to be published. KS made substantial contributions to conception and design, or acquisition of data, or analysis; was involved in drafting the manuscript or revising it critically for important intellectual content; and interpretation of data and gave final approval of the version to be published. NS made substantial contributions to conception and design, or acquisition of data, or analysis and interpretation of data and gave final approval of the version to be published. ST made substantial contributions to conception and design, or acquisition of data, or analysis and interpretation of data and gave final approval of the version to be published. JMC-G made substantial contributions to conception and design, or acquisition of data, or analysis and interpretation of data and gave final approval of the version to be published. RLS made substantial contributions to conception and design, or acquisition of data, or analysis and interpretation of data and gave final approval of the version to be published.

## Pre-publication history

The pre-publication history for this paper can be accessed here:

http://www.biomedcentral.com/1471-2458/10/447/prepub
